# Circulating microRNA-196a as a candidate diagnostic biomarker for chronic hepatitis C

**DOI:** 10.3892/mmr.2015.3386

**Published:** 2015-02-24

**Authors:** BO LIU, YING XIANG, HENG-SHU ZHANG

**Affiliations:** Department of Burns and Plastic Surgery, The First Affiliated Hospital of Chongqing Medical University, Chongqing 400016, P.R. China

**Keywords:** microRNA, microRNA-196a, serum, chronic hepatitis C, biomarker

## Abstract

Previous studies have demonstrated the inhibitory effect of microRNA (miR)-196a on hepatitis C virus (HCV) expression in human hepatocytes. However, the clinical implications of aberrant miR-196a expression and the application of circulating miR-196a in the diagnosis and management of chronic hepatitis C (CHC) require further investigation. The present study aimed to examine the possibility of using serum miR-196a as a biomarker for CHC. The Affymetrix miRNA array platform was used for miRNA expression profiling in adenovirus (Ad)-HCV core-infected (HepG2-HCV) and Ad-enhanced green fluorescence protein (EGFP)-infected HepG2 cells (HepG2-control). miR-196a downregulation and levels were analyzed using stem-loop reverse transcription quantitative polymerase chain reaction (RT-qPCR) analysis of the sera of 43 patients with CHC and 22 healthy controls. A total of six miRNAs were identified as significantly different (≥1.5 fold; P≤0.05) between the two groups. Of note, significant miR-196a downregulation was observed in HepG2-HCV as compared with HepG2-EGFP. Furthermore, as compared with that of the healthy control group, serum miR-196a was demonstrated to be significantly lower in patients with CHC. In addition, analysis of the receiver operating characteristic (ROC) curve for serum miR-196a revealed an area under the ROC curve of 0.849 (95% confidence interval, 0.756–0.941; P<0.001) with 81.8% sensitivity and 76.7% specificity in discriminating chronic HCV infection from healthy controls at a cut-off value of 6.115×10^‒5^, demonstrating significant diagnostic value for CHC. However, no correlation was identified between serum miR-196a and alanine aminotransferase, aspartate aminotransferase or HCV-RNA. In conclusion, the present study identified circulating miR-196a as a specific and noninvasive candidate biomarker for the diagnosis of CHC.

## Introduction

Chronic hepatitis C infection (CHC), which is present worldwide, has been identified to increasingly contribute to health care expenditure, morbidity and mortality (1). Although a strong correlation has been observed between stage and prognosis in CHC, current CHC screening methods have significant limitations (2). Although hepatitis C virus (HCV)-RNA is currently the ‘gold standard’ for the diagnosis of HCV infection and is frequently used for assessing the efficacy of anti-viral agents, HCV viral load does not necessarily accurately correlate with the severity and progression of the disease (3). Thus, the investigation of novel sensitive and specific biomarkers for the early diagnosis of CHC is required.

MicroRNAs (miRNAs) are single-stranded RNAs of endogenous origin with a length of ~22 nucleotides, which function in the post-transcriptional regulation of gene expression (4). This regulation is effected via the mediation of mRNA degradation and/or translational blockade (5); thus, they possess important roles in a variety of physiological and pathological processes (6,7). Of note, miRNAs have been identified as crucial in the pathogenesis of HCV infection-associated liver disease; dysregulations of miRNA have been demonstrated to be involved in the modulation of HCV replication (8–10), translation (11), gene expression (12,13) and in the control of its response to interferon (IFN) (14).

With their stability in circulation, relative convenience of extraction, quantification and detection and the power of polymerase chain reaction (PCR), circulating miRNAs can be used effectively as noninvasive biomarkers (15). miRNAs are used in a wide range of human diseases, including tumors, cardiac injury, tissue injury, sepsis and pregnancy and offer potential for earlier diagnosis, disease progression monitoring and improved precision of personalized medication (15). A previous study observed complementation between miR-196a and the nonstructural (NS) 5A coding region of the HCV JFH1 genome; in addition, IFN-β treatment led to significant miR-196 induction in the Huh-7 human hepatoma cell line and in primary murine hepatocytes (12). This suggested a significant role for miR-196a in the modulation of HCV expression and the therapeutic response of antiviral agents in human hepatocytes. A previous study identified that miR-196a inhibited HCV expression in the HCV replicon cell line and J6/JFH1 HCV cell culture system, in addition to targeting the HCV genome and the 3′-untranslated region of Bach1 mRNA (13). The latter leads to upregulation of the heme oxygenase (decycling) 1 gene, a key cytoprotective enzyme that generates antioxidative and anti-inflammatory molecules (13). Thus, miR-196a may represent an important factor in the pathogenesis of HCV infection. It was suggested that upregulation of miR-196a may be used in a novel strategy to prevent or treat HCV infection, and miR-196a may be valuable in the diagnosis and management of this disease (13). However, the clinical implications of aberrant miR-196a expression and the value of circulating miR-196a in the diagnosis and management of chronic HCV infection require further investigation.

Using an *in vitro* cell culture model and serum samples from clinical patients, the present study aimed to investigate the use of miR-196a as a novel candidate serum biomarker for early CHC diagnosis.

## Materials and methods

### Cell culture

HepG2 cells, purchased from the American Type Culture Collection (Manassas, VA, USA), were cultured in minimum essential medium (GE Healthcare Life Sciences, Logan, UT, USA) supplemented with 10% (v/v) fetal calf serum, 2 mmol/l glutamine, 100 U/ml penicillin and 100 μg/ml streptomycin (all from Gibco Life Technologies, Carlsbad, CA, USA) at 37°C in a humidified chamber.

### Construction of the Ad-HCV core adenovirus and the infection of HepG2 cells

Using the Stratagene AdEasy system (Agilent Technologies, Inc., La Jolla, CA, USA), the Ad-HCV core adenovirus and the control Ad-green fluorescent protein adenovirus were constructed as previously reported (16). The infection of HepG2 cells (at a multiplicity of infection of 50) and the evaluation of the infection efficiency were performed according to the same study (16). Cells were then harvested for miRNA array, total RNA, protein analysis and immunohistochemistry.

### miRNA microarray analysis

miRNA microarray analysis was performed as previously described (17). Briefly, following the extraction of total RNA from the HepG2-HCV and HepG2-control cells using TRIzol (Invitrogen Life Technolodies, Carlsbad, CA), miRNA arrays (Affymetrix, Inc., Santa Clara, CA, USA) were labeled and hybridized according to the manufacturer’s instructions. The comparisons of miRNA expression data between groups were performed with ComparativeMarkerSelection suite in GenePattern software, version 10 (http://www.broadinstitute.org/cancer/software/genepattern).

### Western blot analysis

Proteins extracted by the M-PER Mammalian Protein Extraction Reagent (Cell Signaling Technology, Inc., Danvers, MA, USA) were resolved on 10% SDS-PAGE gels (Bio-Rad Laboratories, Inc., Hercules, CA, USA) and transferred to polyvinylidene fluoride membranes (Pierce Biotechnology, Inc., Rockford, IL, USA). The monoclonal mouse anti-Flag (the Ad-HCV core was tagged with 3X Flag) primary antibody (1:500; ab49763; Abcam, Cambridge, UK) was used overnight at 4°C and the horseradish peroxidase-linked rabbit anti-mouse IgG (1:10,000; ab97046; Abcam) was used at room temperature for 1 h as the secondary antibody. The monoclonal mouse GAPDH antibody (1:1,000; ab8245; Abcam) was used overnight at 4°C as a loading control. Blots were developed using Supersignal WestPico chemiluminescent substrate (Pierce Biotechnology, Inc.), imaged and analyzed using the Bio-Rad ChemiDoc XRS Gel Imaging System (Bio-Rad Laboratories, Inc.).

### Ethics statement

The experiments involving human participation were conducted in accordance with the Declaration of Helsinki of 1975 and were approved by the Medical Ethics Committee on human research of the First Affiliated Hospital of Chongqing Medical University (Chongqing, China). All participants provided written informed consent prior to enrollment.

### Serum collection and storage

Blood samples from the patients in the emergency department were collected, and through a two-step centrifugation (10 min of 820 × g, then 10 min of 16,000 × g at 4°C), the supernatant was transferred to RNase/DNase-free tubes and stored at ‒80°C within 1 h of collection.

### Serum chemistry

Serum levels of alanine aminotransferase (ALT) and aspartate aminotransferase (AST) in patients with CHC and healthy controls were detected using the standard automatic biochemistry analyzer (AU5400; Olympus Corporation, Tokyo, Japan).

### Patient enrollment

Between January 2012 and Feburary 2012, 43 consecutive patients with CHC and 22 healthy volunteers at the First Affiliated Hospital of Chongqing Medical University were recruited. The inclusion criteria for patients with biliary calculi were based on the newly developed universal definition of biliary calculi. Briefly, the patients with biliary calculi were clinically diagnosed by biochemical markers, acute right upper quadrant abdominal colicky pain and detection of calculi by sonography or cholecystography. A total of 28 healthy volunteers with normal liver function and no history of hepatobiliary disease were recruited as non-biliary calculi controls.

### Serum miRNA extraction and stem-loop reverse transcription-quantitative PCR (RT-qPCR)

Using the mirVana PARIS miRNA isolation kit (Ambion Life Technologies, Carlsbad, CA, USA), total RNA enriched with miRNAs was extracted from the serum according to the manufacturer’s instructions. RT-qPCR was conducted in order to determine the expression levels of miR-196a. miRNAs were quantified through the TaqMan miRNA RT-qPCR assay according to the manufacturer’s instructions (Applied BioSystems Life Technologies, Foster City, CA, USA). Briefly, RT-qPCR amplification was performed with gene-specific forward primer and a reverse primer (Applied Biosystems Life Technologies) along with a probe in an ABI Prizm 7500 PCR machine (Applied Biosystems Life Technologies), preceded by first-strand cDNA synthesis with 10 ng RNA and miRNA-196a-specific, stem-loop primer, or U6 stem-loop primer, a control endogenous miRNA (Applied Biosystems Life Technologies). The reverse-transcribed primers were designed as follows: miR-196a, 5′-GTCAGAAGGAATGATGCACAGCCAACAACA-3′; and U6: 5′-AACGCTTCACGAATTTGCGT-3′. The PCR primers were as follows: Mature miR-196a, forward 5′-CGTCAGAAGGAATGATGCACAG-3′, and reverse 5′-ACCTGCGTAGGTAGTTTCATGT-3′; and U6, forward 5′-CTCGCTTCGGCAGCACA-3′, and reverse 5′-AACGCTTCACGAATTTGCGT-3′.

### Relative miRNA expression was calculated from experiments in triplicate following normalization to those for U6 RNA

Relative miR-196a production, reported as 2^‒∆∆Ct^ (Ct represents the threshold cycle), was determined by the ∆Ct method. Differences in miR-196a concentration between the two groups were expressed as fold changes.

### Statistical analysis

Values are presented as the mean ± standard deviation unless otherwise indicated. Spearman correlation analysis, the Mann-Whitney U test, Student’s t-test, or the χ^2^ test was conducted for between-group comparisons as appropriate. The receiver operating characteristic (ROC) curves were established for discriminating patients with CHC from the normal controls. Two-tailed P<0.05 was considered to indicate a statistically significant difference. All statistical calculations were performed using SAS software, version 9.1.3 (SAS Institute, Marlow, UK) and SPSS software, version 17.0 (SPSS, Inc., Chicago, IL, USA).

## Results

### HepG2-HCV and HepG2-control groups exhibit differences in miRNA expression profiles

**Figure 1:**
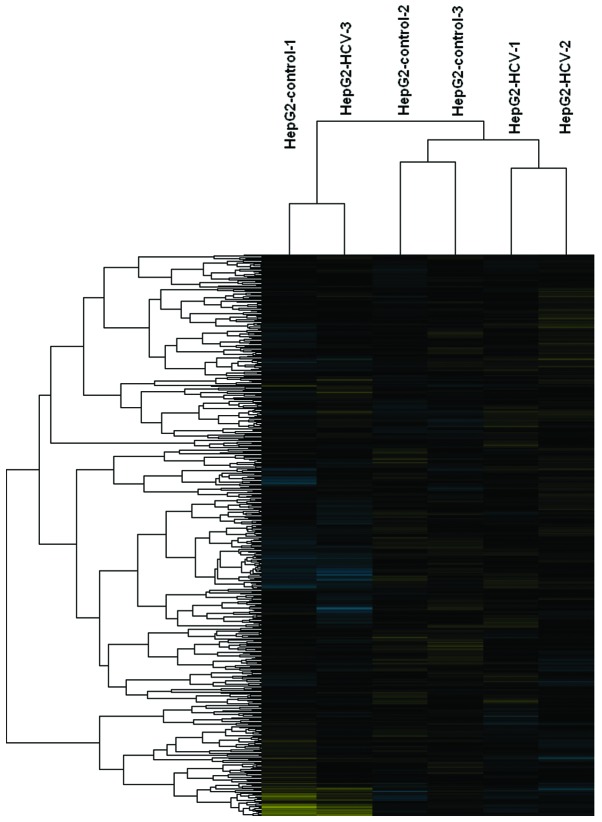
Expression profile of microRNAs in HepG2-hepatitis C virus and HepG2-control cells.

A total of six differentially expressed miRNAs, with a fold-difference ≥1.5 and P≤0.05, were identified between the HepG2-HCV and HepG2-control cells following miRNA microarray analysis (Fig. 1). Among these miRNAs, miR-29a, miR146a, miR-149, miR-221 and miR-222 were identified to be upregulated, while miR-196a was downregulated by the overexpression of the HCV core protein (Table I).

### miR-196a is significantly downregulated in the Ad-HCV infection group

**Figure 2:**
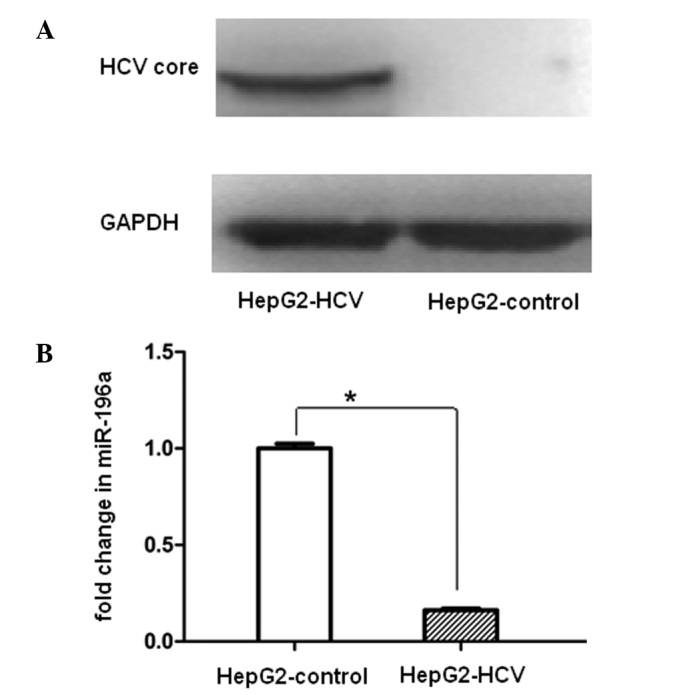
miR-196a was significantly downregulated by Ad-HCV infection. (A) Confirmation of HCV core protein expression using western blot analysis following Ad-HCV infection for 48 h. (B) Relative expression levels of miR-196a in HepG2-HCV and HepG2-control cells. Values are presented as mean ± standard deviation; each experiment was conducted in triplicate. U6 was used as an internal control for miRNA reverse transcription quantitative polymerase chain reaction experiments; ^*^P<0.05. miR, microRNA; HCV, hepatitis C virus.

To investigate whether the miR-196a expression levels were affected by HCV core overexpression, HepG2 cells were infected with Ad-HCV core. miR-196a was significantly downregulated in HepG2-HCV cells as compared with that in the HepG2-control following efficient expression of the HCV core protein at 48 h (Fig. 2).

### Serum miR-196a is significantly reduced in patients with CHC and is diagnostically valuable for CHC

In order to investigate the clinical implications of aberrant miR-196a expression and the use of circulating miR-196 in the diagnosis and management of CHC, sera from 43 patients with CHC and 22 healthy volunteers were collected for biomarker validation. Between-group comparisons of the general clinical characteristics demonstrated that there were no significant differences in the gender ratio and mean age, but significant differences in ALT, AST and HCV-RNA (Table II).

**Figure 3:**
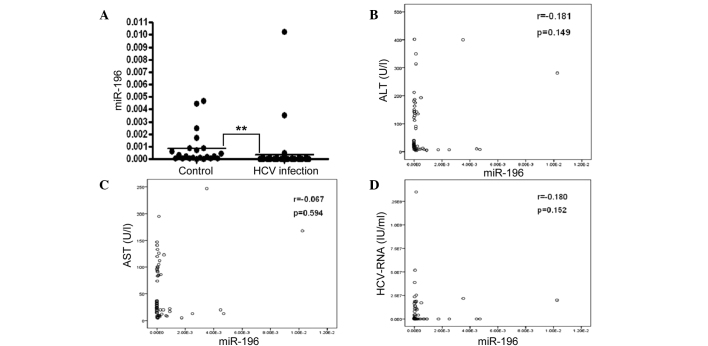
Reduced serum miR-196a levels in patients with CHC. (A) Comparison of miR-196a concentrations between CHC and healthy controls. Serum miR-196a concentrations in correlation with (B) ALT, (C) AST and (D) HCV-RNA. ^**^P<0.001. miR, microRNA; CHC, chronic hepatitis C; ALT, alanine aminotransferase; AST, aspartate aminotransferase; HCV, hepatitis C virus.

Circulating miR-196a was observed to be significantly lower in the CHC group as compared with that in the control group (P<0.001; Fig. 3A). Investigation of the possible correlation between circulating miR-196a levels and the liver injury degree identified no correlation between serum miR-196a and ALT/AST (Fig. 3B and C). Nor was a correlation observed between miR-196a and HCV-RNA (Fig. 3D).

**Figure 4:**
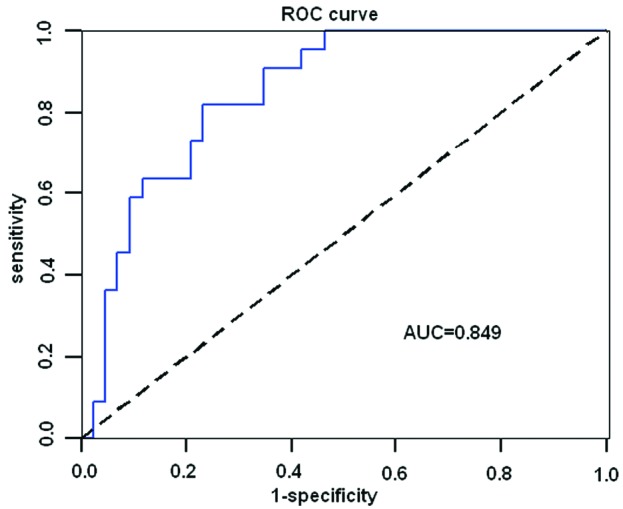
ROC curve analysis of circulating miR-196a levels for discriminating chronic hepatitis C from healthy controls. miR-196a, microRNA-196a; ROC, receiver operating characteristic; AUC, area under the ROC curve.

To further investigate the characteristics of miR-196a as a potential biomarker of CHC, ROC curve analysis was performed. Analysis of the ROC curves for serum miR-196a demonstrated an AUC (area under the ROC curve) of 0.849 (95%CI: 0.756–0.941; P<0.001) with 81.8% sensitivity and 76.7% specificity in discriminating chronic HCV infection from healthy controls at a cut-off value of 6.115×10^‒5^ (Fig. 4). This suggested diagnostic value of circulating miR-196a in CHC.

## Discussion

Due to the absence of reliable and predictive markers for the early diagnosis of HCV infection, treatment for CHC is often delayed. Though HCV viral load analysis has impacted the evaluation of the response likelihood of patients to therapy with PEGylated IFN and ribavirin (18), viral load monitoring is unable to assess the severity of disease or risk of progression, as serum HCV RNA levels remain stable for up to four years (19). The present study confirmed that HCV core protein significantly downregulated miR-196a expression in HepG2 cells. Furthermore, the clinical implications of aberrant miR-196a expression and the use of circulating miR-196 in the diagnosis and management of CHC was validated by the fact that serum miR-196a levels were significantly reduced in patients with CHC. Finally, serum miR-196a levels were identified to be diagnostically valuable for CHC by producing an AUC of 0.849 (95%CI: 0.756–0.941; P<0.001) with 81.8% sensitivity and 76.7% specificity in discriminating CHC from healthy controls at a cut-off value of 6.115×10^‒5^. These results indicated the potential for use of circulating miR-196a as a sensitive and informative biomarker for CHC. However, no correlations were observed between the expression levels of miR-196a, HCV viral load and ALT status.

miR-196 has been previously demonstrated to have critical roles in normal development (20–22) and in the pathogenesis of human malignancy (23–26), immunology, inflammation and virus defense (12,27,28), which has led to various studies attempting to decode its functions. The present study suggested serum miR-196a as a novel biomarker for CHC while profiling miRNAs in HCV core protein-overexpressing HepG2 cells. The observation that the serum miR-196a was relatively low in patients with CHC, but may be easily detected in serum from healthy controls demonstrated for the first time that monitoring of circulating miR-196a may also be applied in clinical CHC diagnosis. ROC analysis identified that miR-196a may be a sensitive, specific and practical clinical diagnostic biomarker for CHC.

Although the present study had a small sample size, it provided the first clinical evidence of the use of circulating miR-196a as a biomarker of CHC, to the best of our knowledge. However, further experiments with a larger sample size are required to extensively evaluate the potential of miR-196a as a practical biomarker. Circulating miRNAs are becoming attractive biomarker candidates and are increasingly used in the prevention, diagnosis, prognosis and therapeutic monitoring of various human diseases (29). By demonstrating that circulating miRNA levels returned to baseline levels following tumorectomy, chemotherapy, acute myocardial infarction recovery and other medical interventions, circulating miRNAs are proving to be promising biomarkers for monitoring therapeutic effects (15). Thus it would be beneficial to monitor the dynamic alterations in plasma miR-196a levels during IFN treatment for CHC. In addition, various previous studies have compared circulating miRNA biomarkers to existing markers and demonstrated a strong correlation in miRNA expression and current marker identification (30–32). Furthermore, Resnick *et al* (33) and Zhu *et al* (34) reported that coupled with additional established markers, circulating miRNAs demonstrate a greater sensitivity than either used alone. Thus, serum miRNA biomarkers in combination with other established biomarkers may provide significant advantages in early diagnosis and prognosis prediction. Therefore, it will be valuable to investigate combined miR-196a and HCV-RNA detection in the assessment of disease severity and progression risk during CHC.

In conclusion, circulating miR-196a was significantly reduced in patients with CHC, potentially via reduced release of miR-196a from HCV-infected hepatocytes. Thus, the presence of reduced circulating miR-196a may be a novel sensitive and specific biomarker for early detection of CHC in humans.

## Figures and Tables

**Table I:** Differentially expressed miRNA profiles between HepG2-HCV and HepG2-control cells.

Expression	miRNAs	Fold change
	miR-29	1.6
	miR-146a	2.0
	miR-149	1.8
	miR-221	1.8
	miR-222	1.5
Downregulated	miR-196a	1.9

miRNA, microRNA; HCV, hepatitis C virus.

**Table II:** Clinical characteristics of the healthy control and chronic hepatitis C group.

Characteristic	Healthy control group (n=22)	Chronic hepatitis C group (n=43)	P-value
Age (mean ± standard deviation)	36.8±9.7	42.0±9.4	<0.05
Male, n (%)	11 (50.0)	27 (62.8)	>0.05
Female, n (%)	11 (50.0)	16 (37.2)	
Alanine aminotransferase (U/l)	9.9±4.9	111.7±107.8	<0.001
Aspartate aminotransferase (U/l)	11.0±5.5	73.0±55.8	<0.001
Hepatitis C virus-RNA (copies/ml)	<1.0×10^3^	>1.0×10^3^	<0.001
